# Nanoporous aramid nanofibre separators for nonaqueous redox flow batteries

**DOI:** 10.1038/s41467-018-05752-x

**Published:** 2018-10-10

**Authors:** Siu on Tung, Sydney L. Fisher, Nicholas A. Kotov, Levi T. Thompson

**Affiliations:** 1 Macromolecular Science and Engineering, University of Michigan, Ann Arbor, MI 48109 USA; 2 Department of Chemical Engineering, University of Michigan, Ann Arbor, MI 48109 USA; 3 Materials Science and Engineering Department, University of Michigan, Ann Arbor, MI 48109 USA; 4 Biomedical Engineering Department, University of Michigan, Ann Arbor, MI 48109 USA; 5 Biointerfaces Institute, University of Michigan, Ann Arbor, MI 48109 USA; 6 Department of Mechanical Engineering, University of Michigan, Ann Arbor, MI 48109 USA; 7 Hydrogen Energy Technology Laboratory, University of Michigan, Ann Arbor, MI 48109 USA

## Abstract

Redox flow batteries are attractive for large-scale energy storage due to a combination of high theoretical efficiencies and decoupled power and energy storage capacities. Efforts to significantly increase energy densities by using nonaqueous electrolytes have been impeded by separators with low selectivities. Here, we report nanoporous separators based on aramid nanofibres, which are assembled using a scalable, low cost, spin-assisted layer-by-layer technique. The multilayer structure yields 5 ± 0.5 nm pores, enabling nanofiltration with high selectivity. Further, surface modifications using polyelectrolytes result in enhanced performance. In vanadium acetylacetonate/acetonitrile-based electrolytes, the coated separator exhibits permeabilities an order of magnitude lower and ionic conductivities five times higher than those of a commercial separator. In addition, the coated separators exhibit exceptional stability, showing minimal degradation after more than 100 h of cycling. The low permeability translates into high coulombic efficiency in flow cell charge/discharge experiments performed at cycle times relevant for large-scale applications (5 h).

## Abstract (web summary)

Nonaqueous redox flow batteries may offer high energy and power densities, but development of separators is key for optimization. Here the authors achieve high coulombic efficiency with a nanoporous aramid nanofibres-based separator with low permeability, high ion conductivity, and exceptional stability.

## Introduction

Redox flow batteries (RFBs) have been identified as promising candidates for grid scale energy storage^1^. The flow batteries offer significant advantages over other large-scale storage options, including flexibility as a consequence of decoupled power and energy densities, long lifetimes, and facile thermal management^2,3^. RFBs store energy in liquid electrolytes that contain redox-active species and supporting electrolytes dissolved in a solvent. During charge and discharge the electrolytes are circulated through porous electrodes on either side of a flow cell assembly^4^. Electrically insulating separators or ion exchange membranes (IEMs) are used to isolate the positive and negative electrolytes while allowing counter ion transport to maintain overall charge neutrality^5^. A highly selective separator or IEM is key to developing economically viable RFBs, as keeping the active species separated is critical to limiting self-discharge and achieving high coulombic efficiencies.

Commercially available RFBs utilize aqueous solutions of transition metal salts^3,6^, however, maximum energy densities for these RFBs are limited by the relatively narrow electrochemical window of water (~1.2–1.6 V)^7–9^. An attractive approach to overcome this limitation involves the use of nonaqueous solvents^10^. Nonaqueous solvents offer improved voltage windows (>4 V)^11^, and with the recent development of highly soluble active species^12,13^, nonaqueous RFBs (NAqRFBs) present an opportunity to increase energy and power densities beyond those of aqueous systems^5,8^. However, one of the major challenges to NAqRFB development is the lack of suitable IEMs or separators. A variety of IEMs are available for use in aqueous RFBs^5,8,14,15^, but they are relatively ineffective in nonaqueous media. For example, the ionic conductivity of Nafion is five orders of magnitude lower for acetonitrile-based electrolytes compared to aqueous electrolytes^16,17^. In addition, a number of promising NAqRFB chemistries use anions such as tetrafluoroborate (BF_4_^−^) or hexafluorophosphate (PF_6_^−^) as charge carriers^9,10,18–23^, necessitating anion-exchange membranes (AEMs). Several AEMs including Neosepta AHA (ASTOM, Japan)^9,10^, UltrexTM AMI-7001 (Membranes international Ltd., USA)^24^, and FAP4 (FuMa-Tech Co.)^22^ have been used in H-type and flow battery configurations. These AEMs, which were designed primarily for water treatment^25^, are plagued by incompatibility with organic solvents^8^. In developing new membranes/separators suitable for use in NAqRFBs, the goal is to design materials that provide high selectivity, allowing facile transport of supporting electrolyte ions while prohibiting the transport of active species. Additionally, this material should exhibit excellent chemical robustness and mechanical properties, such as strength and toughness, along with low manufacturing costs.

Only a few IEMs have been developed specifically for NAqRFBs. Kim et al^26^. described the fabrication of a composite material consisting of a porous polyolefin separator infiltrated with a quaternized poly(styrene-divinylbenzene-vinylbenzyl chloride) copolymer. Maurya et al. synthesized an AEM via the simultaneous polymerization and quaternization of 4-vinyl pyridine and subsequent film casting into a thin membrane^27^. More recently, Won demonstrated an approach for the formation of a separator, coating a porous Celgard 2400 support with poly(diallyldimethylammonium chloride) and urushi composite to form an ion-selective membrane^28^. These IEMs have demonstrated lower permeability of the active species as a result of the cross-linked polymer chains and markedly improved mechanical stabilities. However, concerns have been raised regarding cost and incompatibility with the organic solvents and active materials in NAqRFBs^8^.

In response to these concerns, nanoporous separators have been investigated for use in NAqRFBs. Nanoporous separators achieve selectivity by taking advantage of size differences between the redox-active species and the supporting electrolyte ions. The major advantages associated with these separators are the simplicity of design and low cost when compared to IEMs^29^. Nanoporous silica^30^, polyacrylonitrile^31^, and composite^32^ membranes have been successfully demonstrated in aqueous RFBs. These types of separators have also been demonstrated for polysulfide blocking in lithium–sulfur flow batteries using a polymer with intrinsic microporosity^33^. For NAqRFBs, size-selectivity was recently reported by utilizing electrolytes based on redox-active polymers with sizes on the same order of magnitude as the pores in Celgard 2325 (28 nm), a commercial polyolefin separator^34^. However, many of the most promising active species under consideration for NAqRFBs are significantly smaller in size (<1 nm)^27,33^. For a practical nanoporous separator, pore sizes on the order of a few nanometers are required. Many approaches have been employed to create nanoporous separators, including the use of ordered templates^35^, carbon nanotubes^36^, and various composite nanofibres^37–40^. However, applications are typically for aqueous solutions used in biomolecule separation and water treatment, and are incompatible with organic solvents ^41,42^.

More recently, aramid nanofibres (ANFs) have emerged as a new nanoscale building block^43,44^. Composites prepared from ANF possess biomimetic nanofibre “skeleton” reminiscent of nanofibre networks found in soft tissues have demonstrated superior mechanical properties and high thermal stability^43,45–51^ owing to the parent aramid fibers, well-known under the tradename Kevlar^TM^. Furthermore, ANF composites developed for lithium-ion batteries have demonstrated dendrite-suppressing capabilities due to a combination of high mechanical strength and nanoporosity^48,49,51^. Pore sizes in these materials are tunable and nanopores approaching the sizes required for NAqRFB applications were demonstrated^48,49,51^. The consistently high strength and toughness demonstrated by cartilage-like ANF composites with polymers^52^ could also provide ultrathin separators with minimal resistance.

Here, we explore the feasibility of using ANF-based films as separators for NAqRFBs. Films with pore sizes relevant for use in NAqRFBs are fabricated using a spin-assisted, layer-by-layer (LBL) assembly technique. The permeability, conductivity, stability, and flow cell performance of the ANF separators are compared to those for Celgard 2325 and Neosepta AHA (Neosepta). The surfaces of these ANF separators are coated via LBL with poly(diallyldimethylammonium chloride) and poly(styrene sulfonate) to further increase the selectivity. For the research described in this paper, vanadium acetylacetonate (V(acac)_3_), a well-studied metal coordination complex for NAqRFBs^10,18,23,24,27,53^, is used as a model active species with acetonitrile as the solvent. Although V(acac)_3_ presents stability issues that limit its large-scale implementation^54^, it is selected here due to its commercial availability and more importantly its similar size to a variety of other promising active species, such as metal coordinated complexes^53,55,56^ and organic molecules^13,57–61^, ensuring that the results can be easily translated to more advanced active species systems as they emerge. Overall, this research offers a strategy for the development of size-selective nanoporous separators for more efficient RFBs and presents a surface modification approach that can be used to further enhance the performance of nanoporous separators.

## Results

### Fabrication and structure

A spin-assisted LBL deposition method modified from the original procedure developed by Jiang et al.^62^ was used to fabricate the ANF nanoporous separators. The LBL method is particularly suitable for the preparation of biomimetic high-performance composites with exceptional uniformity, mechanical performance and structural versatility^63,64^. Integration of spin-coating with LBL affords rapid manufacturing, scalability and low cost^65^, while maintaining the uniformity and structural control that LBL provides^66^. In the framework of this study and technological needs of RFBs, uniform micron-scale thickness for minimized resistance and controllable nanometer sized pores required for selectivity are of our particular interest^8,67^.

**Fig. 1:**
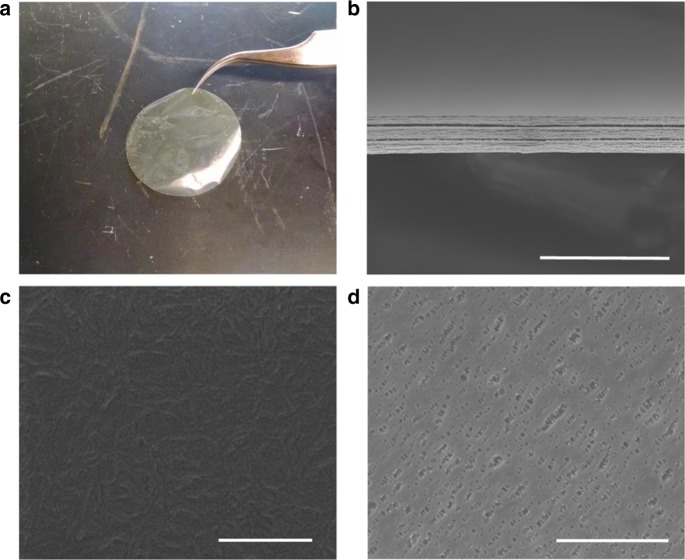
**Images of the ANF and Celgard separators. a** Optical image of a neat aramid nanofibre (ANF) separator. **b** Cross-sectional scanning electron microscopy (SEM) image of a neat ANF separator. **c** SEM image of the surface of a neat ANF separator. **d** SEM image of the surface of Celgard 2325. Scale bars represent 30 μm in (**b**), 500 nm in (**c**) and 3 μm in (**d**)

An 8.5-μm free-standing film was obtained by LBL assembling 20 layers of ANF. Each deposited layer of ANF is estimated to be 425 nm thick. Layers of ANF, or ANF mats, are stacked together to form the separator. This multilayer structure, in conjunction with the dense network of nanofibres comprising each layer, reduces the effective pore size of the separator while maintaining networks of pores for ionic conductivity. The transparency and smoothness illustrated in Fig. 1a indicate the homogeneity of the prepared nanocomposites. The uniform thickness and the homogeneity (8.5 ± 0.2 μm) can be further observed from the scanning electron micrographs (Fig. 1b). Fourier transform infrared (FTIR) spectra of the ANF separator indicate that the molecular structure matches that of previously reported ANF materials (Supplementary Figure 1)^43^. Scanning electron microscopy (SEM) imaging of the separator surface shows a tightly assembled mat of ANFs (Fig. 1c), while results from N_2_ physisorption and mercury intrusion porosimetry (MIP) indicate the presence of pores with average sizes of 5.0 ± 0.5 nm (Supplementary Figure 2). These pores are considerably smaller than those found in Celgard 2325 where the average pore size is 28 nm by MIP and pores as large as 390 nm are found on the surface (Fig. 1d). As a result, the nanoporous ANF separator is expected to significantly reduce the permeability of nanosized active species. The nonporous Neosepta is markedly thicker than Celgard (25 µm) and ANF (8.5 µm) at 192 µm. To quantify the effect of these morphological differences, relevant physical properties, including permeability and conductivity, were measured. Celgard 2325 and Neosepta will be used in this study as comparative benchmarks because of their widespread use in RFBs ^8–10,19,21,34,68^.

### Permeability and conductivity

The permeability of V(acac)_3_ through the separators was determined using an H-type cell. Changes in the V(acac)_3_ concentrations in each of the cell chambers, which were determined using UV–vis spectroscopy, were measured as a function of time. At the start of the experiment, one chamber is filled with 50 mM V(acac)_3_/0.1 M tetrabutylammonium tetrafluoroborate in acetonitrile (TBABF_4_/ACN), while the other chamber is filled with 0.1 M TBABF_4_/ACN. The ANF and Celgard 2325 materials do not appear to interact with V(acac)_3_ as the absorbance peaks (298 nm and 343 nm) are identical (Supplementary Figure 3). The concentrations are obtained by monitoring changes in the absorbance at 298 nm (Supplementary Figure 3 inset). When separated by Celgard 2325, concentrations in the two chambers were nearly equal after 5 h, while the ANF separated chambers took more than 12 h to equilibrate. This significant decrease in the crossover rate is reflected in the permeability of V(acac)_3_ (equation provided in the Methods), which is an order of magnitude lower for ANF than for Celgard 2325 (Table 1). While the reported of size of V(acac)_3_ (9 Å)^8^ is smaller than the pore size of the ANF separators, we believe the tortuosity from the multilayer structure as well as potential Donnan exclusion effects allow the ANF separator to impede the transport V(acac)_3_. For Neosepta, complete equilibration did not occur even after 5 days (120 h), however, later results will demonstrate the incompatibility of Neosepta with acetonitrile.

**Table 1:** Permeability and conductivity of Celgard 2325, Neosepta, aramid nanofibre (ANF) separators and coated ANF separators

Sample	Permeability (×10^−7^ cm^2^ s^−1^)	Conductivity (mS cm^−1^)
Celgard 2325	7.22	0.59
Neosepta AHA	0.03	0.02
ANF	0.82	0.10
(PDDA/PSS)_5_ on ANF	0.007	0.10
(PDDA/PSS)_20_ on ANF	0.003	0.04

For RFB separators, it is important to allow transport of the supporting electrolyte ions while preventing active species crossover. For the purposes of this work, TBABF_4_ is used as the supporting electrolyte and the ionic conductivities of the various separators/membranes are measured using electrochemical impedance spectroscopy. All samples were soaked in a 0.1 M TBABF_4_/acetonitrile solution for 10 days prior to the conductivity measurements. No evidence of swelling is observed for the materials, indicating stability in dimensions. The ANF separator exhibits an ionic conductivity of 0.10 mS cm^−1^, five times lower than that for the Celgard 2325 separator (Table 1). This result is not unexpected given the reduced pore size, which slightly impedes ion mobility.

The ionic conductivity for the ANF separator is five times higher than that for Neosepta and the permeability is an order of magnitude higher (Table 1). This difference is expected given differences in the ionic conducting mechanisms for separators and membranes. For separators such as ANF, ionic conductivity is achieved through the liquid electrolyte impregnated in the nanosized pores (5 nm). For membranes such as Neosepta, ions are conducted in the solid phase via channels formed by charged pendant group on the polymer chains^69^. Therefore, ANF offers a higher ionic conductivity than Neosepta whereas Neosepta offers a lower permeability. We believe that LBL-functionalization of the ANF surface with polyelectrolytes could further reduce the permeability of the associated separator without sacrificing conductivity ^70^.

### Surface modification with polyelectrolytes by LBL assembly

Poly(diallyldimethylammonium chloride) (PDDA), a polycation, and poly(styrene sulfonate) (PSS), a polyanion are used to coat surfaces of the ANF separators. A standard dip-rinse LBL method was used, taking advantage of the ability of this technique to form surfaces of complex geometries^71^ even with nanoscale curvature^72,73^. The application of surface coatings on flow battery membranes has been proposed by Shin et al.^8^, and demonstrated in aqueous RFBs with PDDA/PSS coated Nafion membranes with lowered vanadium ion permeability, and increased coulombic and energy efficiencies^74^. More recently, PDDA/urushi (a natural-occurring polymer commonly seen in Japanese furniture) coatings have been applied to polyolefin microporous separators for NaqRFBs^28^. However, the microporous nature of the polyolefin separators required coating thicknesses on the same order of magnitude as the thickness of the separator to impact permeability and as a result the ionic conductivity was negatively affected. On the other hand, ANF separators with inherent nanoporosity provide an opportunity where only a few layers of PDDA and PSS should be sufficient to affect significant reductions in permeability without significantly impact ionic conductivity. The addition of the charged PDDA/PSS layers on the surface of ANF will also enable the Donnan exclusion effect, where similarly charged species, such as positively charged V(acac)_3_ and PDDA, experience a repulsive effect and therefore lower separator permeability^75^.

PDDA and PSS were coated onto neat ANF using a LBL technique, where a repeated process of 5 min immersion into 1% PDDA, rinse, air dry, 5 min immersion into 1% PSS, rinse, air dry is used to build up layers on the surface of neat ANF. SEM imaging of the coated ANF separator surfaces shows a smooth morphology (Supplementary Figure 4a) in particular when compared to uncoated ANF (Fig. 1c) and energy dispersive spectroscopy (EDAX) shows Na, S, and Cl signals, which are consistent with the deposition of PDDA and PSS (Supplementary Figure 4b). The FTIR spectra of the (PDDA/PSS)_5_ coated ANF, with the subscript denoting five bilayers of PDDA/PSS deposited onto an ANF separator, yield a nearly identical spectrum as the uncoated ANF (Supplementary Figure 1). This indicates that the deposits are relatively thin and do not alter the bulk of the ANF separator, although it is also due in part to the overlap between the identifying structural vibrations for ANF, and PSS and PDDA. For example, sulfonate (–SO_3_) stretching vibrations from PSS overlap with the phenyl–nitrogen vibrations from ANF.

The permeability of V(acac)_3_ through the ANF separator coated with five PDDA/PSS bilayers is two orders lower than that for the uncoated ANF, while the ionic conductivity remains relatively constant at 0.1 mS cm^−1^. The deposition of 20 PDDA/PSS bilayers did not significantly affect the V(acac)_3_ permeability, but the ionic conductivity is reduced by an order of magnitude compared to (PDDA/PSS)_5_ on ANF (Table 1). This indicates that only a thin layer of PDDA/PSS is required to effectively reduce the permeability of the larger V(acac)_3_ molecules while retaining similar transport properties for the smaller BF_4_^−^ ions. When compared to Neosepta, the (PDDA/PSS)_5_/ANF had a ~10 times lower V(acac)_3_ permeability while the conductivity is ~5 times higher. This reduction in permeability without sacrificing conductivity represents a powerful opportunity to increase the efficiencies in RFBs applications without negatively affecting the power density.

### Chemical stability

**Fig. 2:**
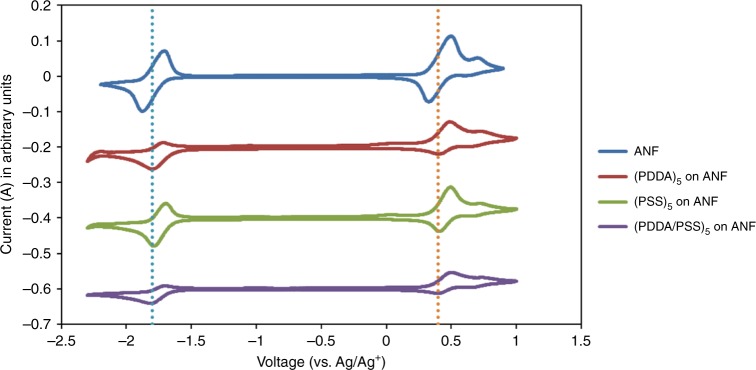
Cyclic voltammagrams of aramid nanofibre (ANF) coated glassy carbon electrodes after soaking in 0.01 M vanadium acetylacetonate/0.1 M tetrabutylammonium tetrafluoroborate in acetonitrile solution for 10 days. Scan rate of 100 mV s^−1^ with the fifth cycle shown

To achieve extended cycling and long lifetimes, the active species, supporting electrolytes and separator materials should not irreversibly interact. Glassy carbon (GC) electrodes are coated with ANF, (PDDA)_5_ on ANF, (PSS)_5_ on ANF and (PDDA/PSS)_5_ on ANF. These electrodes are then soaked in separate 0.01 M V(acac)_3_/0.1 M TBABF_4_/acetonitrile solutions. Cyclic voltammetry (CV) measurements are conducted after 10 days of contact; differences would be an indication of incompatibilities (Fig. 2). The CV for the uncoated ANF showed typical features of V(acac)_3_ with redox potentials at 0.4 V and −1.8 V. The redox couple at 0.6 V, which is attributed to vanadyl acetylacetonate,VO(acac)_2_, is also observed^10^. No new redox activity is observed indicating that ANF is stable within the voltage window of V(acac)_3_ and no interactions between ANF and V(acac)_3_ can be observed electrochemically. Similarly, for the (PDDA)_5_, (PSS)_5_, and (PDDA/PSS)_5_ coated electrodes, no chemical or electrochemical instabilities are observed. This confirms that the components are stable within the operating voltage window of the V(acac)_3_ system. Changes in the peak heights that are observed for the coated electrodes are likely due to a difference in V(acac)_3_ transport characteristics through the PDDA and/or PSS containing films^76,77^. The decreased permeability of V(acac)_3_ through the (PDDA/PSS)_5_ separator also results in a lower total current for the coated electrodes than for uncoated ANF as less V(acac)_3_ is able to reach the GC surface through the coating.

### Charge/discharge experiments

To assess the performance characteristics of the ANF-based separators, flow cells were assembled with the ANF and (PDDA/PSS)_5_ on ANF separators; the results were compared to those for cells incorporating Celgard 2325 and Neosepta. V(acac)_3_ served as the active species in the catholyte and anolyte, and the cells were cycled to 50% state-of-charge (SOC) at ~1 mA cm^−2^ (C/5) to provide sufficient time per cycle to quantify crossover effects and align with operating conditions in commercial applications^78^. Under these conditions, with complete electrolyte separation, V(acac)_3_ is expected to demonstrate stable cycling performance for more than 20 cycles^54^. Crossover is expected to decrease the lifetime due to the irreversible formation of VO(acac)_2_ in the catholyte chamber.

**Fig. 3:**
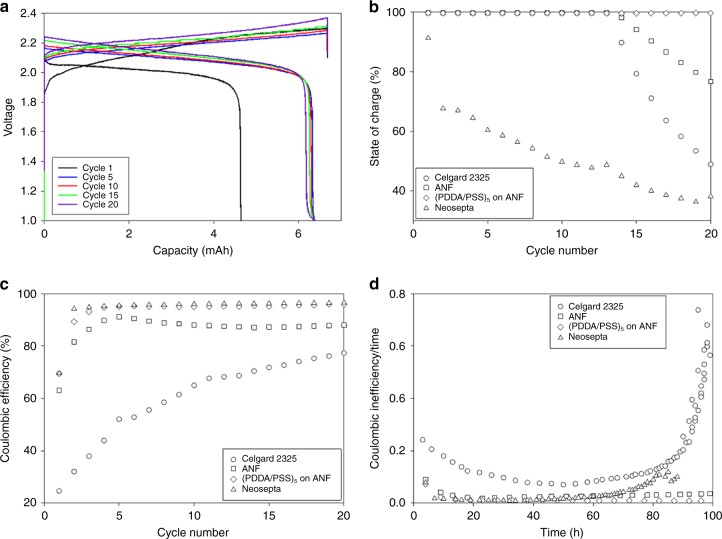
**Comparison of nonaqueous vanadium flow battery performance with ANF and Celgard separators and Neosepta membrane. a** Voltage profiles for an all vanadium acetylacetonate flow cell with poly(diallyldimethylammonium chloride)/poly(styrene sulfonate) (PDDA/PSS)_5_ on aramid nanofibre (ANF) as the separator; **b** normalized charge capacity for Celgard 2325, ANF, (PDDA/PSS)_5_ on ANF and Neosepta; **c** coulombic efficiencies per cycle; and **d** coulombic inefficiency (CIE)/cycle time plotted against total time comparing the degradation rates of each separator/membrane material

Voltage profiles for cells with the (PDDA/PSS)_5_ on ANF separator (Fig. 3a), along with those for the other flow cells (Supplementary Figure 5) reveal an average discharge plateau at 2.2 V. This result is consistent with those from CV (Supplementary Figure 6). Figure 3b shows the charge capacity versus cycle number. For Celgard 2325, the capacity reaches 80% of the initial capacity by cycle 15. The capacity for the ANF separator reaches 80% of the initial capacity by cycle 19. Neosepta shows rapid capacity fade starting with the first cycle and is well below 80% initial capacity by the second cycle. Finally, for the (PDDA/PSS)_5_ on ANF separator, capacity fade is not observed over the 20 cycles, which corresponds to nearly 100 h of continuous stable operation. Furthermore, the (PDDA/PSS)_5_ on ANF separator did not reach 80% capacity until cycle 50 after 220 h of constant cycling (Supplementary Figure 7). Capacity fade for the ANF and Celgard 2325 separators are due to accelerated degradation from V(acac)_3_ crossover. The rapid fade for the cell with Neosepta is likely due to chemical incompatibility, such as the dissolution and subsequent degradation of the active ionomers, rather than crossover.

The coulombic efficiency (CE) of the flow cells with Celgard 2325, Neosepta, ANF and (PDDA/PSS)_5_ on ANF were 55%, 70%, 88%, and 95%, respectively, when averaged over the cycles before reaching 80% of the initial capacity (Fig. 3c). The 95% CE observed with (PDDA/PSS)_5_ on ANF represents one of the highest values reported for separators or membranes designed for NAqRFBs to date (Supplementary Table 1). Similarly, high voltaic efficiencies were observed for Celgard, ANF, and (PDDA/PSS)_5_ on ANF (82-87%), while that for Neosepta was 76%. This reduction is likely due to its thickness (220 µm) and poor conductivity (Table 1). The cells employing ANF or coated ANF demonstrate impressive efficiencies, especially when considering the length of the cycle (~5 h). While Neosepta exhibited a fairly high CE, it also showed the most dramatic capacity fade, suggesting incompatibility with the nonaqueous media. The CE for Celgard 2325 is low due to its high porosity, and trends upwards over the 20 cycles because of continuous capacity fade. Since crossover is a zero-order time-dependent process, the amount of crossover in a single cycle decreases with shortened cycle times.

## Discussion

To account for the time contribution on the CE and better understand the crossover and degradation effects, the coulombic inefficiency (CIE = 1-CE) per cycle time was calculated. This parameter has been used by Smith et al.^79^ to compare parasitic reaction rates in lithium-ion batteries at different charging currents. The CIE/cycle time metric can be thought of as a zero-order cell degradation rate. For example, a crossover process with negligible degradation would be a zero-order reaction, and therefore exhibit a constant CIE/cycle time versus time. Any change in slope, or nonlinear behavior is an indication that additional degradation processes are occurring. While this metric cannot be used to identify the cause of degradation, it is useful in comparing the degradation rates for different cell materials. Over a total of 100 h of cycling, the (PDDA/PSS)_5_ on ANF separator exhibited the lowest degradation rates followed by Neosepta, ANF and Celgard 2325 (Fig. 3d). Higher degradation rates in the first two cycles are likely due to electrode equilibration processes, analogous to the formation of solid electrolyte interphases for lithium-ion batteries^80^. After the first two cycles, the (PDDA/ANF)_5_ on ANF was the only material to exhibit a constant CIE/cycle time over the entire duration of the experiment, which is expected for a simple crossover process with negligible active species degradation. This is also in agreement with the minimal capacity fade observed in Fig. 3a, b. The ANF-based cell exhibited elevated CIE/cycle time values compared to those for the (PDDA/PSS)_5_ on ANF-based cell, which is expected due to its higher permeability. During stable cycling with ANF(<55 h), the CIE/cycle time remains constant, however, it starts to gradually increase once the fade starts. This suggests that several degradation processes are now occurring, including crossover and V(acac)_3_ degradation. The Neosepta and Celgard 2325 cells both are subject to a rapid increase in CIE/cycle time (degradation rate) after 80 h, likely due to chemical incompatibilities for Neosepta and significant crossover for Celgard 2325. These observations suggest complex degradation behavior and demonstrate that these commercial materials are not suitable for use in NAqRFBs.

For an all-V(acac)_3_ flow cell, the oxidation of V(acac)_3_ to VO(acac)_2_ in the catholyte during cycling has been previously reported^10,54^. VO(acac)_2_ has a single reversible redox “wave” at 0.6 V versus Ag/Ag^+^^54^, so no capacity fade will be observed if the degraded material remains in the catholyte chamber. However, any VO(acac)_2_ that crosses to the anolyte chamber becomes inaccessible during charge/discharge. For the higher permeability separators (Celgard 2325 and to some degree, ANF), degradation to VO(acac)_2_ leads to capacity fade due to the loss of active material. This is partially responsible for the dramatic increase in degradation rate observed for Celgard 2325 in Fig. 3d. Conversely, for the (PDDA/PSS)_5_ on ANF separator, active species in the catholyte and anolyte remain relatively isolated and therefore a balanced number of redox events are maintained even after VO(acac)_2_ degradation occurs in the catholyte. In this case, the cycle life is limited only by the stability of VO(acac)_2_ and V(acac)_3_.

**Fig. 4:**
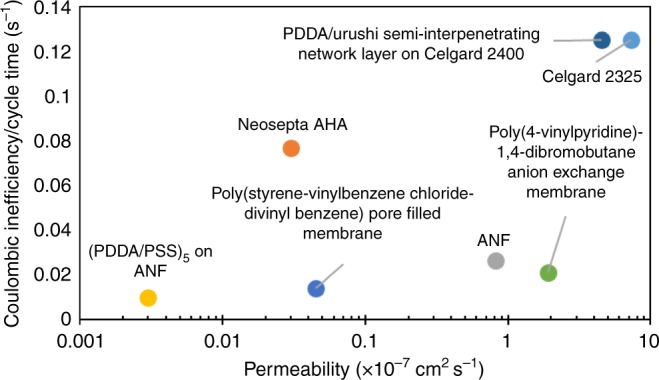
Comparative evaluation of separator/membrane permeability and coulombic inefficiency (CIE)/cycle time degradation rates observed in flow cell charge/discharge experiments between aramid nanofibre (ANF) separators and other nonaqueous redox flow battery separators and membranes. Vanadium acetylacetonate was used as the active species in all cases with the exception of ref. [26] with pore-filled membrane, which used metal bipyridine

The benefits of decreasing separator permeability are summarized in Fig. 4, where separator/membrane permeability and CIE/cycle time data from published reports and our work are compared. A general trend of lower permeability leading to lower CIE/cycle time degradation rates can be observed. Additional CIE/cycle time data for other commercial separators/membranes (reported without permeability) can be found in Supplementary Table 1. Among all surveyed works, (PDDA/PSS)_5_ on ANF exhibited the lowest permeability and CIE/cycle time degradation rate, representing a breakthrough in NAqRFB separator and membrane design. It should be noted that while most of the work represented in the figure utilized V(acac)_3_ in ACN as the model active species, Kim et al.^26^ used tris(2,2′-bipyridyl) complexes of iron and nickel in propylene carbonate as the active species. Despite this major difference in cell chemistry and solvent, the permeability and CIE/cycle time data correlated well with the other data points and illustrates that permeability-CIE/cycle time relationship stands regardless of the cell chemistry used. Furthermore, the asymptotic trend in Fig. 4 illustrates that while continued reduction in permeability can reduce crossover-related degradation, active material reversibility and stability is an eventual limiting factor to improving NAqRFB cycle life. As more stable redox-active materials emerge, we believe even longer operating times for (PDDA/PSS)_5_ on ANF flow cells can be achieved.

In this paper, we describe the fabrication of a nanoporous size-selective separator based on ANF using a spin-assisted LBL. Surface modification of the ANF separators using a standard dip-rinse-dry LBL with polyelectrolytes PDDA and PSS is also described; this modification further decreases the V(acac)_3_ permeability of the separator. The permeability of V(acac)_3_ and the conductivity of TBABF_4_ supporting electrolyte in acetonitrile are measured and compared to those for Celgard 2325, a commercially available polyolefin separator and Neosepta AHA, a commercially available AEM. The LBL-modified ANF separator exhibited an order of magnitude lower permeability due to the small pore size (5 ± 0.5 nm). Surface modification of the ANF separator demonstrated additional reductions in permeability by two orders of magnitude with minimal impact on ionic conductivity. Flow cell charge/discharge studies with commercially relevant cycling times (5 h) verified the reduced permeability for ANF and (PDDA/PSS)_5_ on ANF based on the high Coulombic efficiencies (95%). This represents, to the best of our knowledge, one of the highest CE reported for membranes/separators designed for NAqRFBs. The exceptional stability exhibited by ANF and (PDDA/PSS)_5_ on ANF over 220 h of continuous operation is a testament to the stability of ANF in NAqRFB environments. We envision that with development of highly stable active materials and further optimization of the ANF separators, highly efficient and longer lasting NAqRFBs can be achieved. Established mechanical properties of the membranes stemming from those found in nature inspired LBL composites and aramid-based fibers contribute to their long-term performance and will facilitate their scalable production.

## Methods

### Preparation of aramid nanofibre separators

A 1 wt% ANF dispersion is prepared following the protocol developed by Yang et al.^43^. Glass slides are cleaned by a piranha solution (70:30 wt% H_2_SO_4_: H_2_O_2_) for 4 h and subsequently rinsed with deionized (DI) water extensively. For the neat ANF separators, 1 mL of the ANF dispersion is spin coated onto a glass slide at 1000 rpm for 30 s. The coated glass slide is then dipped into a water bath to remove the dimethyl sulfoxide (DMSO) and potassium hydroxide (KOH) from the dispersion, thus forming a thin ANF hydrogel on the glass. The sample is then dried at 70 °C for 30 min. This process is repeated 20 times in order to build up an 8.5 µm film. Free-standing samples are obtained by chemically etching the glass slide using 0.5% HF solution. The samples are washed extensively in ethanol and then DI water until the rinse water pH is neutral.

### Polyelectrolyte LBL deposition

1% poly(diallyldimethylammonium chloride) solution (20 wt% in water, average molecular weight 400,000–500,000, Sigma Aldrich) and 1% poly(styrene sulfonate) solution (average molecular weight ~1,000,000, Sigma Aldrich) are prepared using DI water. ANF separators are first dipped in the 1% PDDA for 5 min, rinsed in DI water for 1 min, air dried and then dipped into the 1% PSS for 5 min. The sample then again rinsed in DI water and air dried. This cycle is repeated for 5 times for (PDDA/PSS)_5_ on ANF and 20 times for (PDDA/PSS)_20_ on ANF. The last layer in the process is PDDA. Free-standing samples are then obtained using the same glass etching procedures as the neat ANF samples. All PDDA/PSS coated samples are soaked in 0.1 M TBABF_4_ acetonitrile solution for at least 10 days before testing. This is to remove the Na^+^ and Cl^−^ ions from the samples.

### Ionic conductivity experiments

Ionic conductivity is determined using electrochemical impedance spectroscopy (EIS). 20 mm diameter samples are soaked in 0.1 M TBABF_4_ ( ≥ 99.0%, Sigma Aldrich) in acetonitrile (ACN) (99.9 + %, Extra Dry, AcroSeal^TM^, ACROS Organics^TM^), solution for at least 72 h before measurement. The samples are assembled into an EL-Cell (ECC-Std, EL-CELL® GmbH) with 100 µL 0.1 M TBABF_4_ in ACN. The assembly is allowed to equilibrate for 30 min before a measurement. EIS results are fitted with a basic semicircle fit, where the high-frequency intercept is taken as the solution resistance. The solution resistance is used, along with electrode area and sample thickness, to calculate the ionic conductivity.

### Vanadium acetylacetonate permeability experiments

The samples are assembled into an H-Cell setup (Adam & Chittenden Scientific Glass). Totally, 7 mL of 0.05 M V(acac)_3_ (Strem Chemical Inc.) and 0.1 M TBABF_4_/ACN solution is placed in one compartment of the cell and 7 mL of 0.1 M TBABF_4_/ACN solution is placed in the other compartment. The cell is constantly stirred with micro magnetic stir bars. The V(acac)_3_ concentration in the pure supporting electrolyte side is monitored over time using UV–vis spectroscopy. The permeability of the separator is then calculated using the equation below,

1 $$V\frac{{\mathrm{d}}C_t}{{\mathrm{d}}t} = A\frac{P}{L}\left( {C_0 - C_t} \right)$$

where *C*, *V*, *A*, *L*, and *P* are the concentration, volume of the cell, separator area, separator thickness, and V(acac)_3_ permeability of the sample, respectively.

### Aramid nanofibre stability experiments

The tip of a GC electrode (area 0.07 cm^2^, BASi) is coated with a 1% ANF dispersion. The GC electrode is then dipped into water to remove the DMSO and KOH, leaving a layer of ANF hydrogel on the tip. The samples are then dried at 80 °C overnight to remove any water. PDDA, PSS and PDDA/PSS are later added on top of the dried ANF coating using a LBL technique. The coated electrodes are soaked in 0.1 M TBABF_4_ in ACN solution for at least 72 h before use. At the start of the stability measurement, the coated electrodes are inserted into a three-electrode electrochemical cell with a 0.01 M V(acac)_3_ and 0.1 M TBABF_4_ in ACN electrolyte. The electrode and solution are kept in contact for 10 days before the CV is conducted.

### Charge/discharge experiments

Custom flow cells with interdigitated graphite flow fields and polypropylene backing plates are used for all cycling experiments^81^. Carbon paper is used as the electrode (SGL Group, 29 AA), with two pieces inserted on either side of Neosepta, Celgard 2325, ANF, or coated ANF. Polypropylene gasket tape (GORE PTFE Sealant, Gallagher Fluid Seals Inc.) is used to seal the cell at ~20% electrode compression. During cycling, two glass 10 mL reservoirs are filled with electrolyte (0.05 M V(acac)_3_/0.5 M TBABF_4_/ACN), and the fluid is pumped through the cell using a peristaltic pump at 10 mL min^−1^. The cell is cycled at C/5 (~1 mA/cm^2^) with voltage cutoffs at 1.4 and 2.2 V, and a coulombic limit of 80% SOC. The voltage cutoffs were determined from the cyclic voltammogram of V(acac)_3_.

## Electronic supplementary material

Supplementary Information

## Data Availability

The data described in this paper are available from the authors upon request.
