# Supplementary material for: Nanoporous aramid nanofibre separators for nonaqueous redox flow batteries
Source: Nat Commun. 2018 Oct 10;9:4193. doi: 10.1038/s41467-018-05752-x (PMC6180111; doi:10.1038/s41467-018-05752-x)
Supplement: Supplementary file 1 — Supplementary Information [file 41467_2018_5752_MOESM1_ESM.pdf]

Nanoporous Aramid Nanofiber Separators for Non-Aqueous Redox Flow Batteries

Tung et al.

Supplementary Information

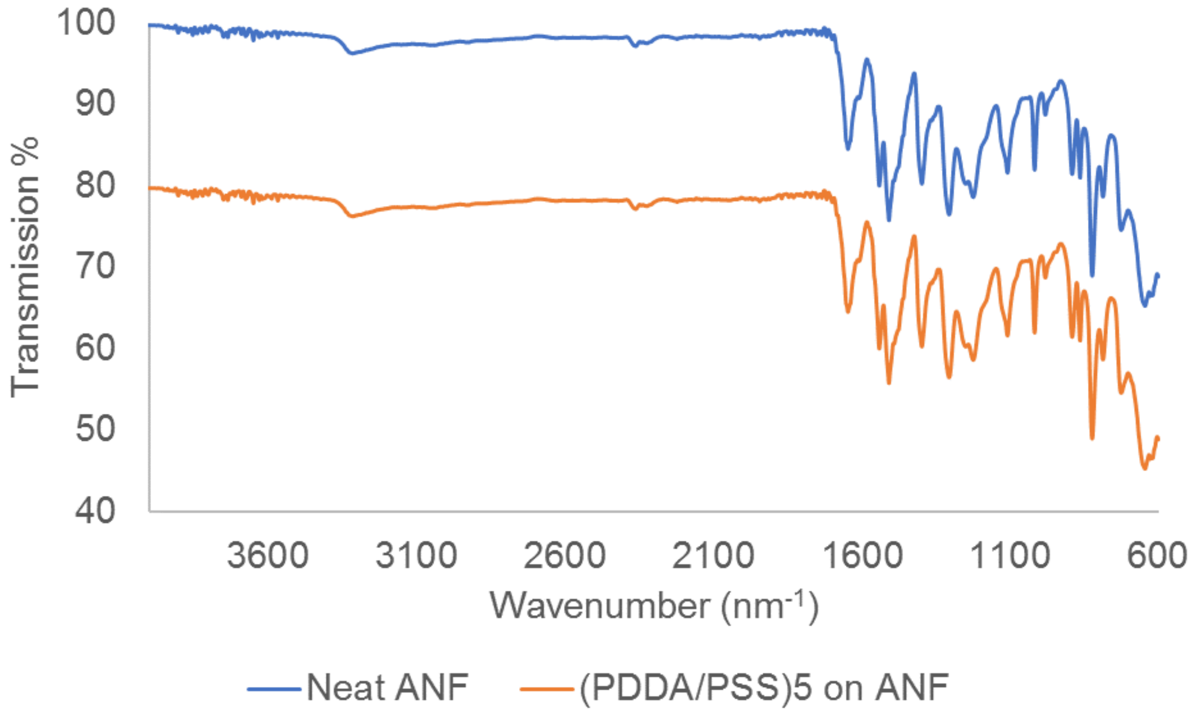

Supplementary Figure 1 FTIR spectra of neat ANF and $(PDDA/PSS)_5$ on ANF

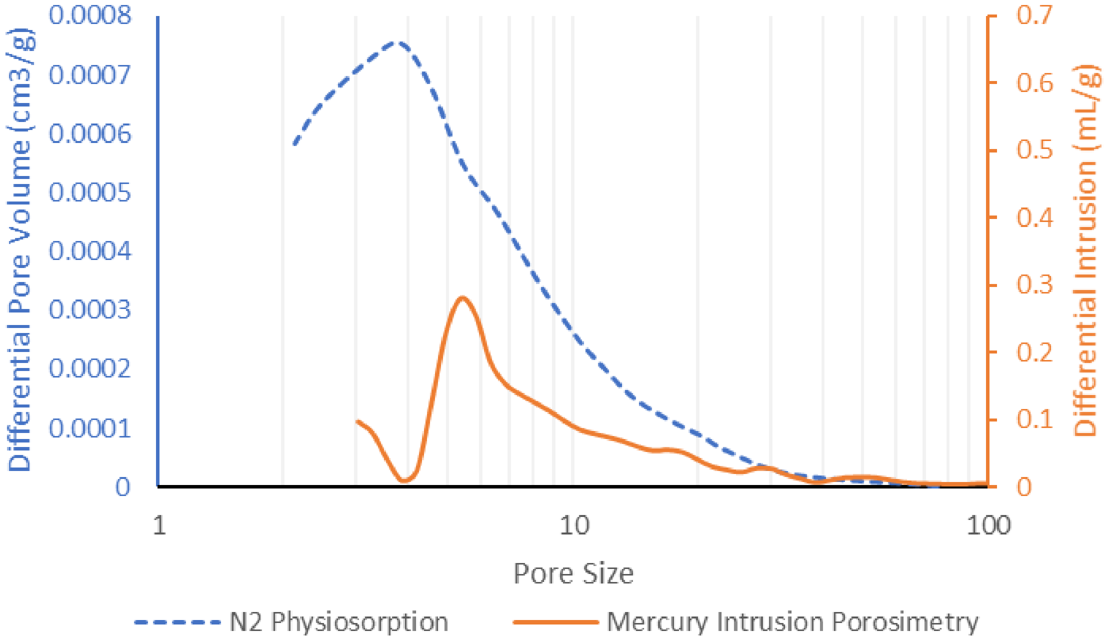

Supplementary Figure 2 Pore size distribution of ANF separator determined using (a) N_2 Physiosorption and (b) mercury intrusion porosimetry

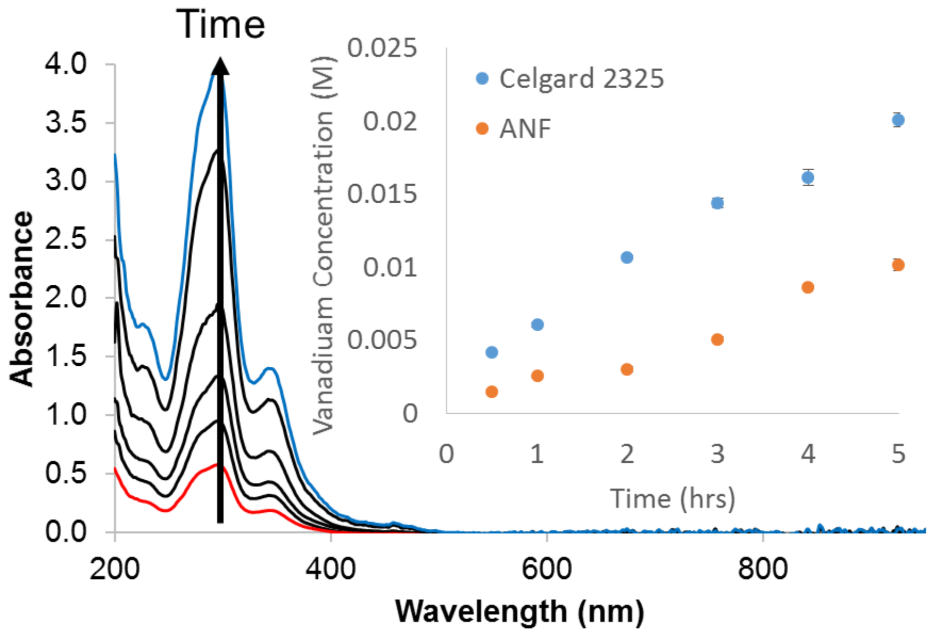

Supplementary Figure 3 UV-Vis spectra of $V(acac)_3$ crossover in Celgard 2325 H-Type cell; Insert: Concentration over time curve of Celgard 2325 and ANF. Neosepta and $(PDDA/ANF)_5$ on ANF did not show any crossover after 5 hours. Error bars are calculated using the standard deviation of 5 measurements.

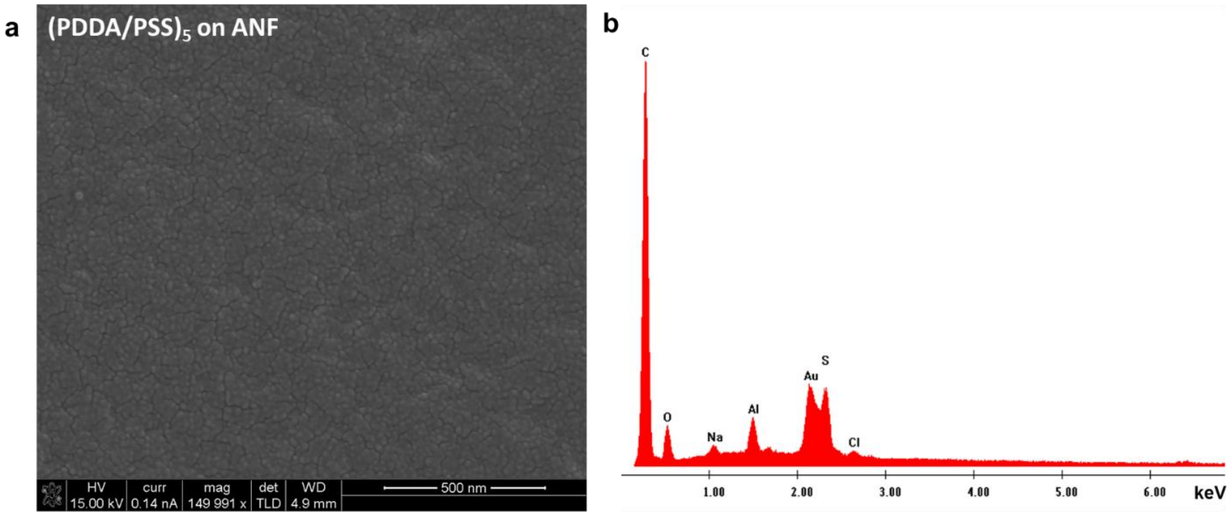

Supplementary Figure 4 (a) Scanning electron micrograph of the surface of $(PDDA/PSS)_5$ on ANF; (b) Energy dispersive spectra (EDAX) of area depicted in (a) showing Na, Cl and S signals confirming the deposition of PDDA and PSS.

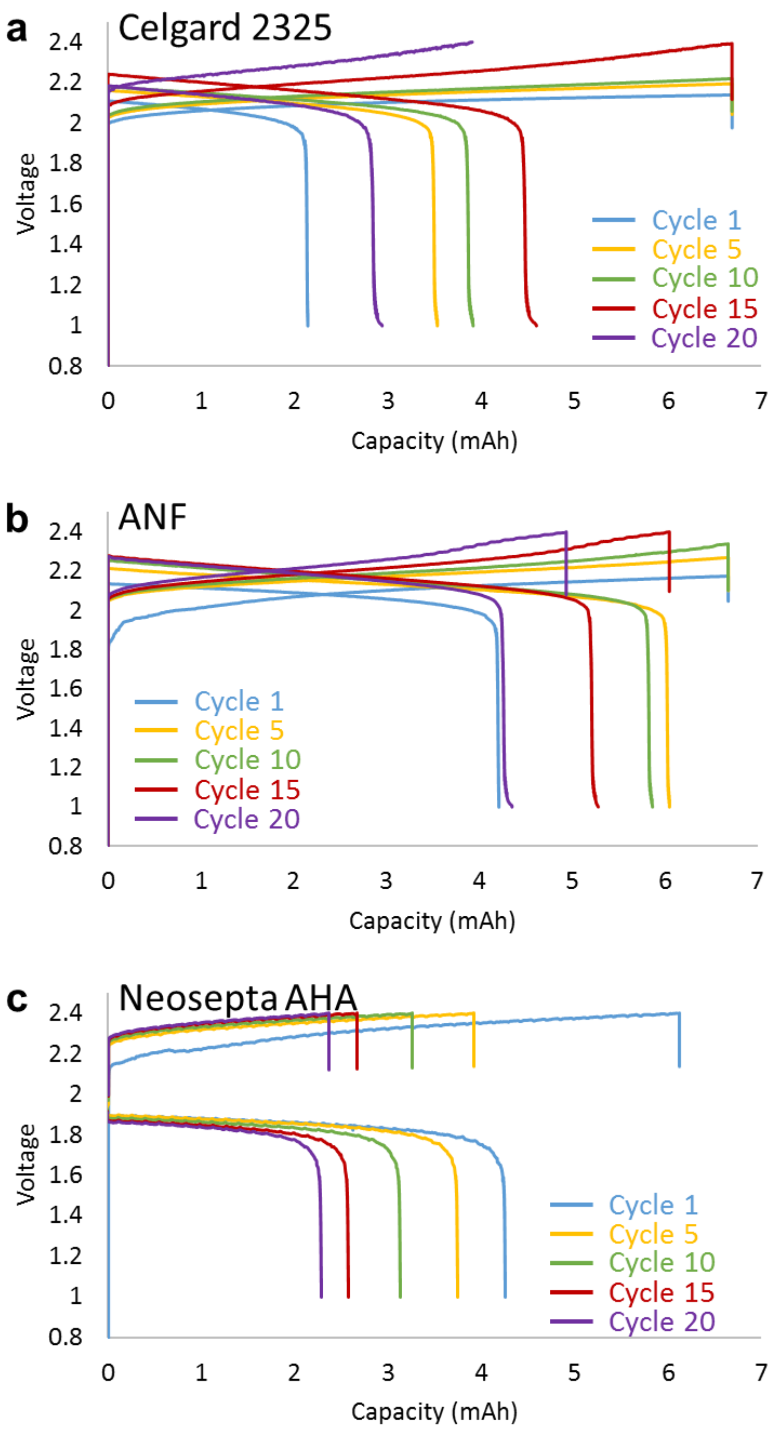

Supplementary Figure 5 Voltage profile of flow cells with (a) Celgard 2325, (b) ANF, and (c) Neosepta AHA as RFB separator.

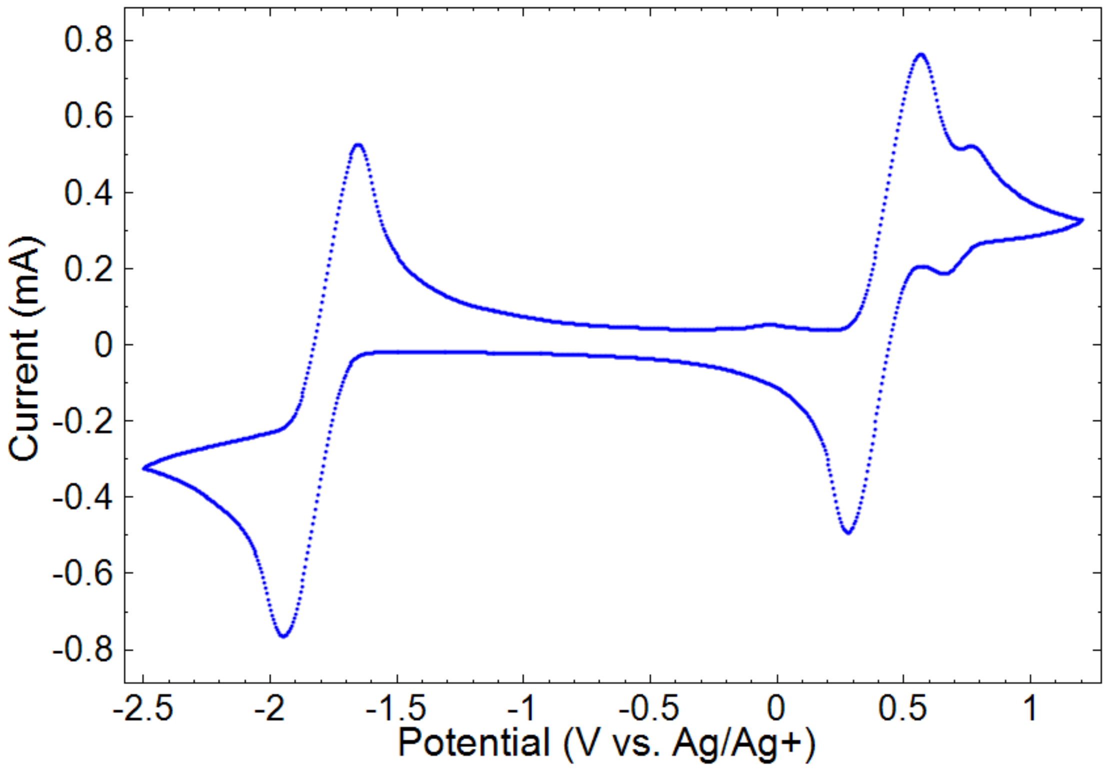

Supplementary Figure 6 Cyclic voltammogram of 10mM $V(acac)_3$, 500mM $TBABF_4$ in acetonitrile; scan rate 100mV/s; 5th cycle shown.

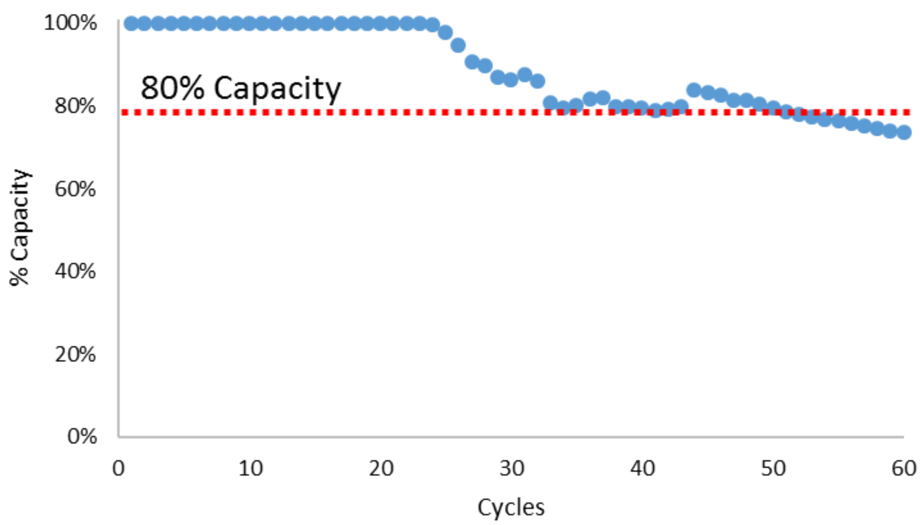

Supplementary Figure 7 % Capacity vs. Cycle for $(PDDA/PSS)_5$ on ANF flow cell. 0.05M $V(acac)_3$ 0.5M $TEABF_4$ in ACN.

Supplementary Table 1 Summary comparing CE and VE of Flow Cells with various membranes

Separator/Membrane	Permeability ($x10^{-7}$ cm^2s^{-1})	Electrolyte Condition	Current Density (mA/cm^2)	Cycle Time (hrs)	Coulombic Efficiency (CE)	Voltaic Efficiency (VE)	CIE/Time (s^{-1})	Reference
Celgard 2325	7.22	0.05M $V(acac)_3$/0.5M $TEABF_4$ in MeCN	1	~3.6	55%#	83%#	0.13	This work
Neosepta AHA	0.03	0.05M $V(acac)_3$/0.5M $TEABF_4$ in MeCN	1	~3.9	70%#	76%#	0.077	This work
ANF	0.82	0.05M $V(acac)_3$/0.5M $TEABF_4$ in MeCN	1	~4.5	88%#	87%#	0.027	This work
$(PDDA/PSS)_5$ on ANF	0.003	0.05M $V(acac)_3$/0.5M $TEABF_4$ in MeCN	1	~4.9	95%#	82%#	0.010	This work
TEA^+-Nafion	-	0.1M $V(acac)_3$/0.5M $TEABF_4$ in MeCN	10	~1.3*	91%	88%	0.069*	[1]
Daramic 175 SLI Microporous Separator	-	0.1M $V(acac)_3$/0.5M $TEABF_4$ in MeCN	10	~1.2*	74%	92%	0.22*	[1]
Neosepta AHA	-	0.1M $V(acac)_3$/0.5M $TEABF_4$ in MeCN	1	~10*	51%	51%	0.049*	[1]
Poly(styrene-vinylbenzene chloride-divinyl benzene) Pore-filled Membrane	0.045	0.4M Fe(bpy)3$(BF_4)_2$/0.2M $Ni(bpy)_3(BF_4)_2$ with 0.5M $TEABF_4$ in PC	-	~3.5*	95%	92%*	0.014*	[2]
Poly(4-vinylpyridine)-1,4-dibromobutane Anion Exchange Membrane	1.91	0.01M $V(acac)_3$/0.1M $TEABF_4$ in MeCN	0.1	~3.8*	92%	96%	0.021*	[3]
PDDA/urushi Semi-interpenetrating Network Layer coated on Celgard 2400	4.53	0.01M $V(acac)_3$/0.1M $TEABF_4$ in MeCN	0.5	~2.4*	70%	61%*	0.13*	[4]

#Averaged over cycles before 80% initial capacity
*Estimated from reported experimental conditions & data
- Value was not given in article

Supplementary References

1. Escalante-Garcia, I. L., Wainright, J. S., Thompson, L. T. & Savinell, R. F. Performance of a Non-Aqueous Vanadium Acetylacetonate Prototype Redox Flow Battery: Examination of Separators and Capacity Decay. *Journal of the Electrochemical Society* 162, A363–A372 (2014).

2. Kim, D. H. *et al.* Pore-filled anion-exchange membranes for non-aqueous redox flow batteries with dual-metal-complex redox shuttles. *Journal of Membrane Science* 454, 44–50 (2014).

3. Maurya, S., Shin, S. H., Sung, K. W. & Moon, S. H. Anion exchange membrane prepared from simultaneous polymerization and quaternization of 4-vinyl pyridine for non-aqueous vanadium redox flow battery applications. *Journal of Power Sources* 255, 325–334 (2014).

4. Cho, E. & Won, J. Novel composite membrane coated with a poly(diallyldimethylammonium chloride)/urushi semi-interpenetrating polymer network for non-aqueous redox flow battery application. *Journal of Power Sources* 335, 12–19 (2016).
